# Supplementary material for: Intent to Be Vaccinated against COVID-19 in Victoria, Australia
Source: Vaccines (Basel). 2022 Jan 28;10(2):209. doi: 10.3390/vaccines10020209 (PMC8878999; doi:10.3390/vaccines10020209)
Supplement: Supplementary file 1 [file vaccines-10-00209-s001.zip › vaccines-1529149-supplementary.pdf]

SUPPLEMENTARY MATERIAL

Table S1. Summary of the proportion of participants exhibiting vaccine intention or lack of vaccine intention prior to being vaccinated.

	…been vaccinated since enrolment	…have answered 'definitely yes' to Q1 at least once	…have answered 'definitely yes' or 'probably yes' to Q1 at least once	…have answered 'definitely not' to Q1 at least once	…have answered 'definitely not' or 'probably not' to Q1 at least once
**Proportion of all participants that have…**	130/686 (19%)	469/686 (68%)	528/686 (77%)	14/686 (2%)	47/686 (7%)
**Proportion of vaccinated participants that have…**	130/565 (23%)	387/565 (69%)	421/565 (75%)	6/565 (1%)	27/565 (5%)
*** Proportion of vaccinated participants, excluding those vaccinated since enrolment, that have…**	NA	387/435 (89%)	421/435 (97%)	6/435 (1%)	27/435 (6%)

*Participants vaccinated since enrolment were excluded from the denominator because it cannot be inferred if vaccine intention was present prior to vaccine uptake. See Table 1 in main text for survey question specification.

Table S2. Summary of participant characteristics as of 9 November 2021.

Demographic	Number	% total (/686)
**Age**		
18-24	125	18%
25-34	143	21%
35-44	96	14%
45-54	107	16%
55-64	117	17%
65+	125	18%
**Healthcare workers**		
Yes	146	21%
No	540	79%
**Chronic health condition increasing risk of COVID-19 ***		
Yes	181	26%
No	505	74%
**Country of birth**		
Australia	476	69%
Other	210	31%
**Highest level of education obtained**		
Primary school	33	5%
High school	93	14%
Tertiary education - postgraduate	187	27%
Tertiary education – TAFE/trade certificate	110	16%
Tertiary - undergraduate	263	38%
**Participant has children living in their household**		
Yes	181	26%
No	505	74%
**Language spoken at home**		
English	606	88%

Other	80	12%
**Employment status pre-COVID-19 (March 2020)**		
Full-time	224	33%
Part-time or casual	242	35%
Self-employed	31	5%
Retired	94	14%
Unemployed	62	9%
Other (including full-time carers)	33	5%
**Gender identity**		
Female	497	72%
Male	189	28%
**Active member of a religious group/church**		
Yes	74	11%
No	612	89%
**Area of residence in Victoria**		
Metropolitan Melbourne	550	80%
Regional Victoria	136	20%

* A binary variable was inferred denoting presence of one or more chronic health conditions that put the participant at increased risk of severe COVID-19. Participants were included in this category if they reported Alzheimer's/dementia, asthma, autoimmune disease, bowel disease, cancer, chronic kidney conditions, chronic lung conditions, diabetes, heart disease, hypertension/high blood pressure, immune disorders, liver conditions, musculoskeletal conditions or stroke.

Table S3. Testing of intention to be vaccinated against COVID-19 ('Already vaccinated' or 'Definitely yes' = 1; all other responses = 0) by demographic group between 1 October 2021 to 21 August 2021. Time periods were excluded where there were <25 participants unwilling to be vaccinated.

Demographic variable	*p*-value ($\chi 2$)
**1 October 2020 to 27 October 2020**	
Age	0.33
Healthcare workers	0.23
Chronic health condition	0.38
Country of birth	0.23
Highest level of education obtained	0.19
Children in the household	0.42
Language spoken at home	0.72
Employment status (pre-COVID-19)	0.54
Gender identity	1.00
Religious	0.54
Area of residence	1.00
**28 October 2020 to 23 November 2020**	
Age	0.60
Healthcare workers	0.10
Chronic health condition	0.39
Country of birth	0.78
Highest level of education obtained	0.07
Children in the household	p<0.01
Language spoken at home	1.00
Employment status (pre-COVID-19)	0.09
Gender identity	0.57
Religious	1.00
Area of residence	0.98
**24 November 2020 to 20 December 2020**	

Age	0.31
Healthcare workers	0.79
Chronic health condition	0.77
Country of birth	0.64
Highest level of education obtained	0.12
Children in the household	p<0.01
Language spoken at home	0.40
Employment status (pre-COVID-19)	0.41
Gender identity	0.77
Religious	0.72
Area of residence	0.92
**21 December 2020 to 16 January 2021**	
Age	0.15
Healthcare workers	1.00
Chronic health condition	0.19
Country of birth	0.38
Highest level of education obtained	0.06
Children in the household	0.20
Language spoken at home	0.52
Employment status (pre-COVID-19)	0.19
Gender identity	0.54
Religious	0.98
Area of residence	0.94
**17 January 2021 to 12 February 2021**	
Age	0.25
Healthcare workers	0.64
Chronic health condition	0.69
Country of birth	1.00
Highest level of education obtained	0.25
Children in the household	0.01
Language spoken at home	1.00
Employment status (pre-COVID-19)	0.23
Gender identity	0.93
Religious	1.00
Area of residence	0.11
**13 February 2021 to 11 March 2021**	
Age	0.08
Healthcare workers	0.22
Chronic health condition	1.00
Country of birth	0.08
Highest level of education obtained	p<0.01
Children in the household	0.22
Language spoken at home	0.77
Employment status (pre-COVID-19)	0.24
Gender identity	0.50
Religious	0.74
Area of residence	0.26
**12 March 2021 to 7 April 2021**	
Age	p<0.01
Healthcare workers	0.07
Chronic health condition	0.17
Country of birth	0.49

Highest level of education obtained	0.65
Children in the household	p<0.01
Language spoken at home	1.00
Employment status (pre-COVID-19)	0.02
Gender identity	0.70
Religious	0.80
Area of residence	0.75
**8 April 2021 to 4 May 2021**	
Age	p<0.01
Healthcare workers	p<0.01
Chronic health condition	0.07
Country of birth	0.89
Highest level of education obtained	0.01
Children in the household	0.11
Language spoken at home	0.94
Employment status (pre-COVID-19)	0.03
Gender identity	0.82
Religious	0.58
Area of residence	0.62
**5 May 2021 to 31 May 2021**	
Age	p<0.01
Healthcare workers	p<0.01
Chronic health condition	0.04
Country of birth	0.33
Highest level of education obtained	0.01
Children in the household	0.01
Language spoken at home	0.80
Employment status (pre-COVID-19)	0.02
Gender identity	0.26
Religious	0.55
Area of residence	0.87
**1 June 2021 to 27 June 2021**	
Age	p<0.01
Healthcare workers	0.03
Chronic health condition	p<0.01
Country of birth	0.35
Highest level of education obtained	p<0.01
Children in the household	0.13
Language spoken at home	1.00
Employment status (pre-COVID-19)	p<0.01
Gender identity	0.65
Religious	0.72
Area of residence	0.21
**28 June 2021 to 24 July 2021**	
Age	p<0.01
Healthcare workers	0.01
Chronic health condition	p<0.01
Country of birth	0.98
Highest level of education obtained	p<0.01
Children in the household	0.27
Language spoken at home	0.35
Employment status (pre-COVID-19)	0.02

Gender identity	1.00
Religious	0.94
Area of residence	1.00
**25 July 2021 to 20 August 2021**	
Age	p<0.01
Healthcare workers	0.03
Chronic health condition	0.02
Country of birth	0.91
Highest level of education obtained	0.04
Children in the household	0.01
Language spoken at home	1.00
Employment status (pre-COVID-19)	p<0.01
Gender identity	0.93
Religious	0.82
Area of residence	1.00

Table S4. Coefficients and corresponding *p*-values for Generalised Estimating Equation (GEE) analysis of intention to vaccinated against COVID-19 ('Already vaccinated' or 'Definitely yes' = 1; all other responses = 0). The reference category is aged 18-24; not a healthcare worker; primary school education; no children in household. Time is treated a continuous variable with four-week increments, with 1 October 2021 to 27 October 2021 = 1.

Coefficient	Estimate	Standard error	*p*-value
Intercept	-0.19	0.47	0.68
Time	0.12	0.02	p<0.01
Age (25-34)	-0.52	0.41	0.20
Age (35-44)	-0.75	0.51	0.14
Age (45-54)	-0.09	0.49	0.86
Age (55-64)	-0.19	0.43	0.66
Age (65+)	0.08	0.49	0.87
Healthcare worker (yes)	-0.22	0.40	0.59
Education - High school	0.20	0.45	0.66
Education - tertiary, postgraduate	0.56	0.42	0.18
Education - tertiary, TAFE/trade	-0.36	0.41	0.38
Education - tertiary, undergraduate	0.45	0.39	0.25
Children in household (yes)	-0.87	0.35	0.01
Time:Age (25-34)	0.04	0.04	0.23
Time:Age (35-44)	0.08	0.04	0.03
Time:Age (45-54)	0.05	0.04	0.22
Time:Age (55-64)	0.08	0.03	0.02
Time:Age (65+)	0.09	0.05	0.05
Time:Healthcare worker (yes)	0.09	0.04	0.03
Time:Children in household (yes)	0.05	0.03	0.05

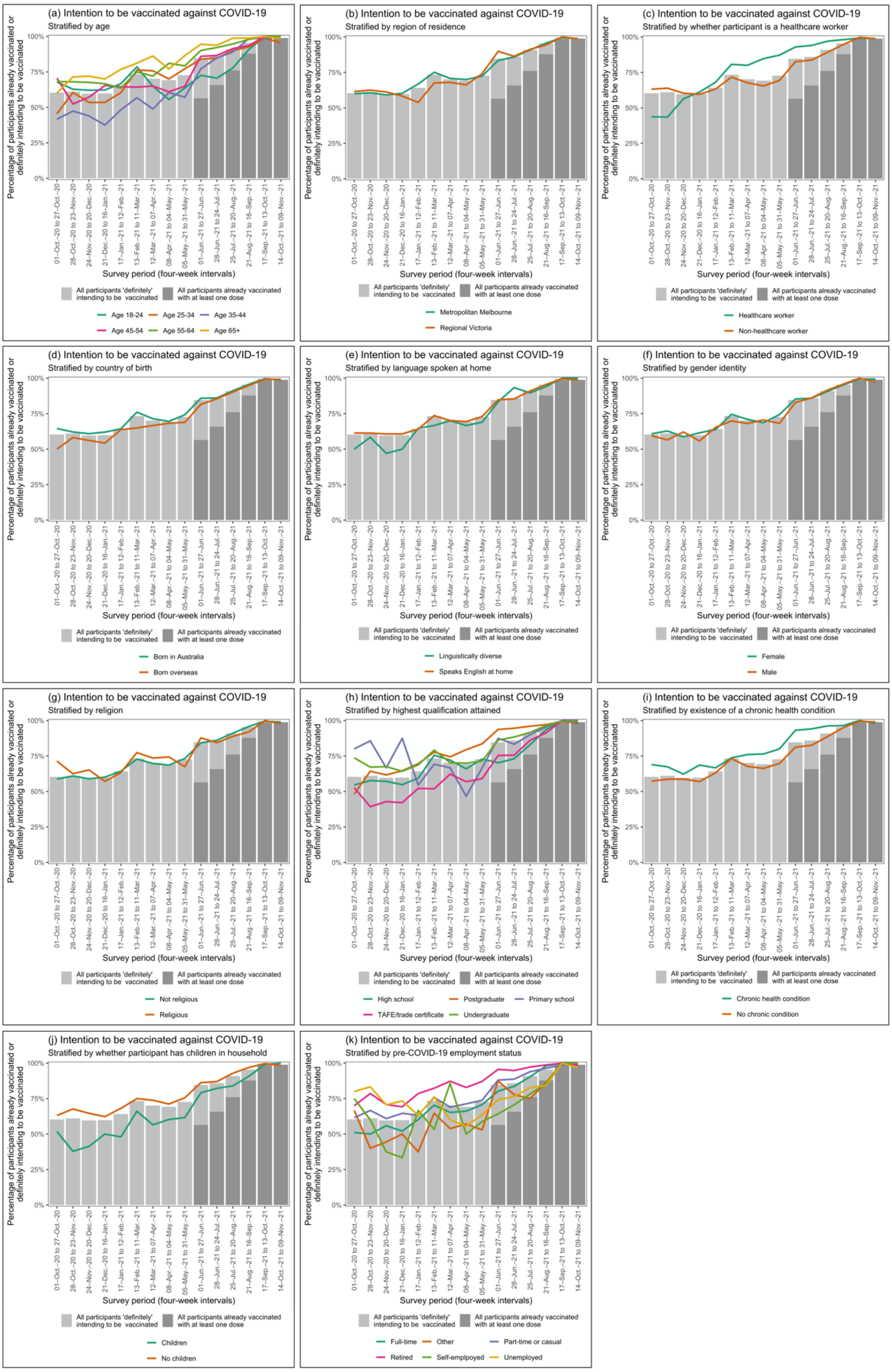

Figure S1. Intent to vaccinate over time, stratified by demographic variables. Grey bars display vaccine intention in all survey respondents, separated by those responding 'Definitely yes' and 'I have already been vaccinated' to Q1 (see Table 1 in main text for survey question specification). Note that data on vaccine uptake are presented from 1 June 2021 onwards only due to the introduction of survey questions assessing vaccine status in May 2021 (see Table 1). Coloured lines indicate the proportion intending to vaccinate by demographic.

Table S5. Testing of acceptance of mandatory vaccination for high-risk groups ('Totally acceptable' = 1; all other responses = 0) by demographic group between 1 October 2021 to 9 November 2021.

Demographic variable	*p*-value ($\chi 2$)
**1 October 2020 to 27 October 2020**	
Age	0.31
Healthcare workers	0.82
Chronic health condition	1.00
Country of birth	0.07
Highest level of education obtained	0.21
Children in the household	0.96
Language spoken at home	1.00
Employment status (pre-COVID-19)	0.24
Gender identity	0.12
Religious	0.75
Area of residence	0.24
**28 October 2020 to 23 November 2020**	
Age	0.66
Healthcare workers	0.63
Chronic health condition	0.57
Country of birth	0.27
Highest level of education obtained	0.20
Children in the household	0.03
Language spoken at home	0.13
Employment status (pre-COVID-19)	0.15
Gender identity	0.24
Religious	0.42
Area of residence	0.89
**24 November 2020 to 20 December 2020**	
Age	0.15
Healthcare workers	0.15
Chronic health condition	0.97
Country of birth	0.11
Highest level of education obtained	0.25
Children in the household	0.03
Language spoken at home	0.60
Employment status (pre-COVID-19)	0.21
Gender identity	0.05
Religious	0.07
Area of residence	0.49
**21 December 2020 to 16 January 2021**	
Age	0.86
Healthcare workers	1.00
Chronic health condition	0.35
Country of birth	1.00
Highest level of education obtained	0.17
Children in the household	$p<0.01$
Language spoken at home	0.30
Employment status (pre-COVID-19)	0.78
Gender identity	0.92
Religious	0.10
Area of residence	0.61

**17 January 2021 to 12 February 2021**	
Age	0.04
Healthcare workers	0.89
Chronic health condition	0.21
Country of birth	0.16
Highest level of education obtained	0.82
Children in the household	0.08
Language spoken at home	0.61
Employment status (pre-COVID-19)	0.78
Gender identity	0.83
Religious	0.62
Area of residence	0.42
**13 February 2021 to 11 March 2021**	
Age	0.39
Healthcare workers	0.18
Chronic health condition	0.17
Country of birth	0.11
Highest level of education obtained	0.31
Children in the household	0.85
Language spoken at home	0.92
Employment status (pre-COVID-19)	0.65
Gender identity	0.03
Religious	1.00
Area of residence	0.93
**12 March 2021 to 7 April 2021**	
Age	0.31
Healthcare workers	0.13
Chronic health condition	0.10
Country of birth	0.56
Highest level of education obtained	0.10
Children in the household	0.07
Language spoken at home	0.29
Employment status (pre-COVID-19)	0.88
Gender identity	0.03
Religious	0.94
Area of residence	0.98
**8 April 2021 to 4 May 2021**	
Age	$p<0.01$
Healthcare workers	0.67
Chronic health condition	0.04
Country of birth	0.07
Highest level of education obtained	0.29
Children in the household	0.03
Language spoken at home	$p<0.01$
Employment status (pre-COVID-19)	0.45
Gender identity	0.48
Religious	0.71
Area of residence	0.64
**5 May 2021 to 31 May 2021**	
Age	$p<0.01$
Healthcare workers	1.00

Chronic health condition	p<0.01
Country of birth	0.69
Highest level of education obtained	0.21
Children in the household	p<0.01
Language spoken at home	0.10
Employment status (pre-COVID-19)	0.04
Gender identity	0.16
Religious	0.21
Area of residence	0.18
**1 June 2021 to 27 June 2021**	
Age	0.09
Healthcare workers	0.80
Chronic health condition	0.15
Country of birth	0.07
Highest level of education obtained	0.15
Children in the household	0.07
Language spoken at home	0.20
Employment status (pre-COVID-19)	0.93
Gender identity	0.06
Religious	0.78
Area of residence	0.51
**28 June 2021 to 24 July 2021**	
Age	p<0.01
Healthcare workers	0.87
Chronic health condition	p<0.01
Country of birth	0.12
Highest level of education obtained	0.14
Children in the household	0.04
Language spoken at home	0.86
Employment status (pre-COVID-19)	p<0.01
Gender identity	0.04
Religious	1.00
Area of residence	0.23
**25 July 2021 to 20 August 2021**	
Age	p<0.01
Healthcare workers	1.00
Chronic health condition	0.32
Country of birth	0.06
Highest level of education obtained	0.15
Children in the household	0.13
Language spoken at home	0.52
Employment status (pre-COVID-19)	p<0.01
Gender identity	0.25
Religious	0.71
Area of residence	0.83
**21 August 2021 to 16 September 2021**	
Age	p<0.01
Healthcare workers	0.85
Chronic health condition	p<0.01
Country of birth	0.54
Highest level of education obtained	0.35
Children in the household	0.02

Language spoken at home	1.00
Employment status (pre-COVID-19)	0.01
Gender identity	0.38
Religious	0.17
Area of residence	0.99
**17 September 2021 to 13 October 2021**	
Age	0.03
Healthcare workers	0.69
Chronic health condition	p<0.01
Country of birth	0.09
Highest level of education obtained	0.05
Children in the household	p<0.01
Language spoken at home	0.67
Employment status (pre-COVID-19)	0.21
Gender identity	0.06
Religious	0.82
Area of residence	0.05
**14 October 2021 to 9 November 2021**	
Age	p<0.01
Healthcare workers	0.57
Chronic health condition	p<0.01
Country of birth	0.02
Highest level of education obtained	0.41
Children in the household	0.07
Language spoken at home	p<0.01
Employment status (pre-COVID-19)	p<0.01
Gender identity	p<0.01
Religious	0.98
Area of residence	0.26

Table S6. Coefficients and corresponding *p*-values for Generalised Estimating Equation (GEE) analysis of acceptance of mandatory vaccination for high-risk groups ('Totally acceptable' = 1; all other responses = 0). The reference category is aged 18-24; male; primary school education. Time is treated a continuous variable with four-week increments, with 1 October 2021 to 27 October 2021 = 1.

Coefficient	Estimate	Standard error	*p*-value
Intercept	0.41	0.41	0.31
Time	-0.03	0.02	0.10
Age (25-34)	-0.55	0.36	0.12
Age (35-44)	-1.00	0.40	0.01
Age (45-54)	-0.41	0.41	0.32
Age (55-64)	-0.64	0.38	0.10
Age (65+)	-0.31	0.41	0.45
Gender (Female)	0.36	0.13	p<0.01
Education - High school	-0.39	0.39	0.31
Education - tertiary, postgraduate	-0.18	0.36	0.61
Education - tertiary, TAFE/trade	-0.73	0.37	0.04
Education - tertiary, undergraduate	-0.31	0.34	0.37
Time:Age (25-34)	0.08	0.03	p<0.01
Time:Age (35-44)	0.12	0.03	p<0.01
Time:Age (45-54)	0.08	0.03	0.01
Time:Age (55-64)	0.14	0.03	p<0.01

Time:Age (65+)	0.12	0.03	p<0.01

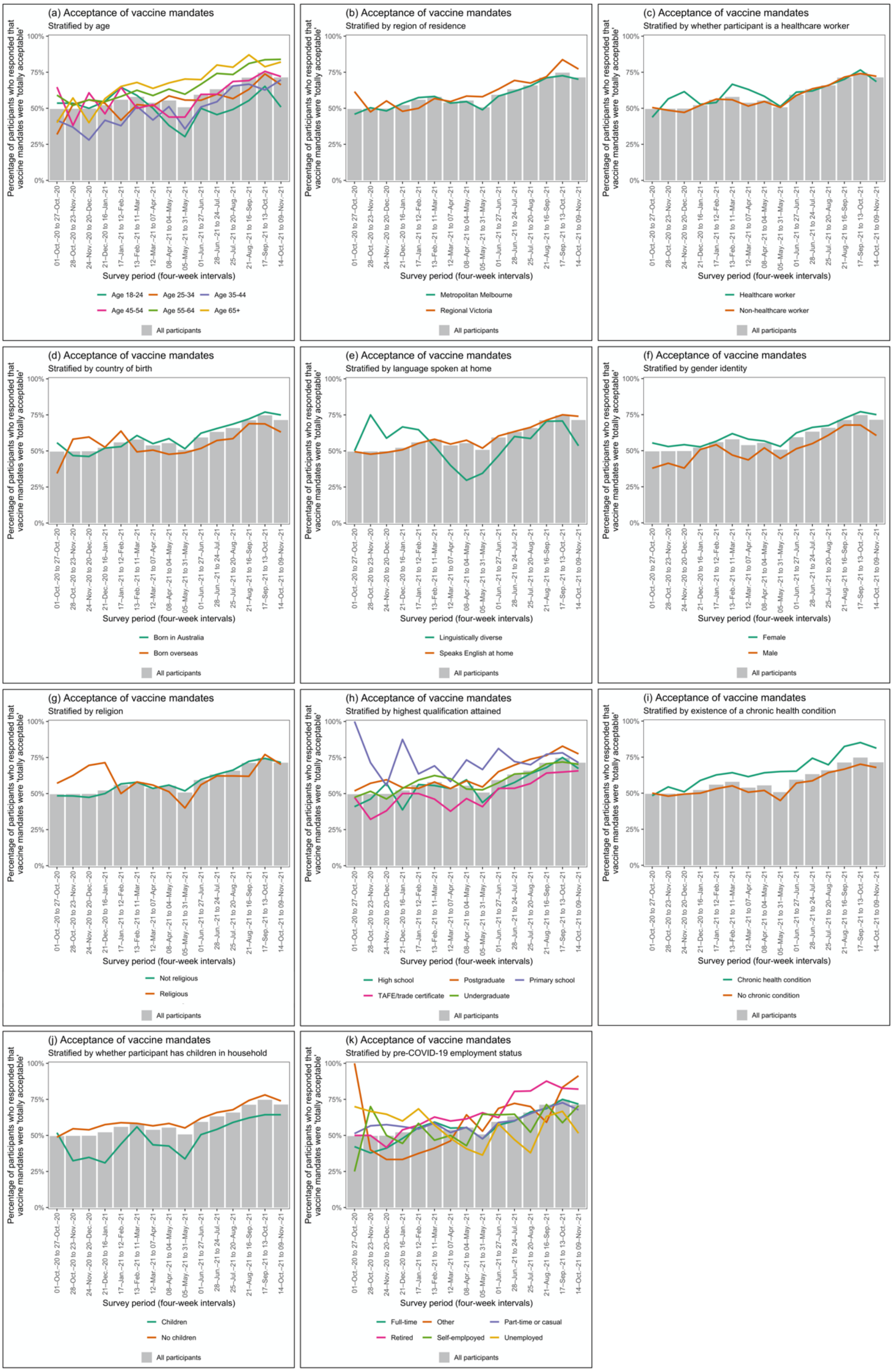

Figure S2. Acceptance of vaccine mandates over time, stratified by demographic variables. Grey bars indicate the proportion of participants responding that vaccine mandates are ‘totally acceptable’ to Q3 (see Table 1 in main text for question wording). Coloured lines indicate the proportion accepting vaccine mandates by demographic.

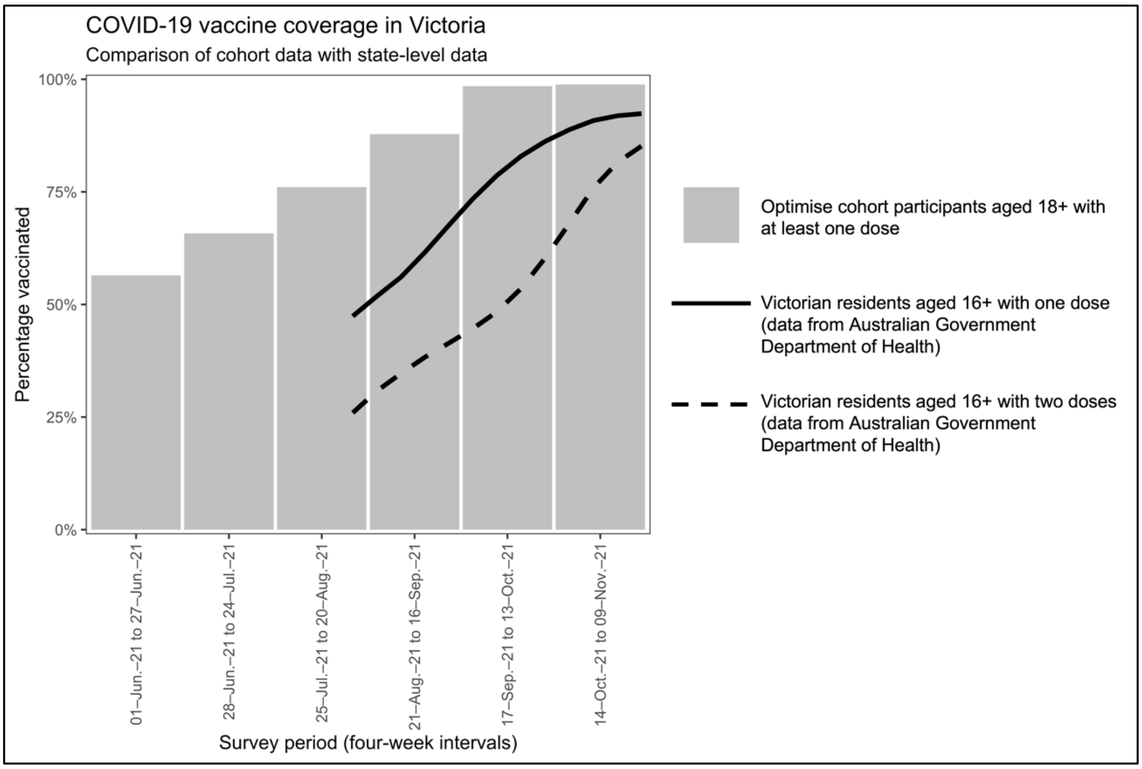

Figure S3. Comparison of COVID-19 vaccine coverage in the study cohort to coverage in the general population in Victoria. State-level vaccine coverage data were obtained from daily reports from the Australian Government Department of Health [1] from the first publication date (16 August 2021) onwards.

References for Supplementary Material

1. Australian Government Department of Health COVID-19 vaccination daily jurisdictional breakdown Available online: https://www.health.gov.au/resources/collections/covid-19-vaccination-daily-jurisdictional-breakdown (accessed on Jan 8, 2021).
